# Study of the Incorporation of Ladle Furnace Slag in the Manufacture of Cold In-Place Recycling with Bitumen Emulsion

**DOI:** 10.3390/ma13214765

**Published:** 2020-10-26

**Authors:** Juan María Terrones-Saeta, Francisco Javier Iglesias-Godino, Francisco Antonio Corpas-Iglesias, Carmen Martínez-García

**Affiliations:** Department of Chemical, Environmental, and Materials Engineering, Higher Polytechnic School of Linares, University of Jaen, Scientific and Technological Campus of Linares, 23700 Linares, Spain; figodino@ujaen.es (F.J.I.-G.); facorpas@ujaen.es (F.A.C.-I.); cmartin@ujaen.es (C.M.-G.)

**Keywords:** ladle furnace slag, reclaimed asphalt pavements, cold in-place recycling, simple compressive strength, bitumen emulsion, waste, circular economy

## Abstract

Cold in-place recycling with bitumen emulsion is a good environmental option for road conservation. The technique produces lower CO_2_ emissions because the product is manufactured and spread in the same location as the previous infrastructure, and its mixing with bitumen emulsion occurs at room temperature. Adding materials with cementitious characteristics gives the final mixture greater resistance and durability, and incorporating an industrial by-product such as ladle furnace slag (of which cementitious characteristics have been corroborated by various authors) enables the creation of sustainable, resistant pavement. This paper describes the incorporation of ladle furnace slag in reclaimed asphalt pavements (RAP) to execute in-place asphalt pavement recycling with bitumen emulsion. Various test groups of samples with increasing percentages of emulsion were created to study both the density of the mixtures obtained, and their dry and post-immersion compressive strength. To determine these characteristics, the physical and chemical properties of the ladle furnace slag and the reclaimed asphalt pavements were analyzed, as well as compatibility with the bitumen emulsion. The aforementioned tests define an optimal combination of RAP (90%), ladle furnace slag (10%), water (2.6%), and emulsion (3.3%), which demonstrated maximum values for compressive strength of the dry and post-immersion bituminous mixture. These tests therefore demonstrate the suitability of ladle furnace slag for cold in-place recycling with bitumen emulsion.

## 1. Introduction

The construction industry is one of the sectors with the greatest environmental impact [1], as well as being crucial for the development of a region’s social welfare and economy. Civil constructions contribute to the progress of a population and the economic development of a nation. Building such constructions is therefore essential, even if large amounts of materials are consumed and the environment is affected [2,3].

A high proportion of the materials consumed by the construction of civil infrastructure projects are natural aggregates from nearby quarries. Taken together, the stages of extracting these natural aggregates and the binder used, transporting them to the factory, and designing the asphalt mix, generate a significant emission of greenhouse gases and a high environmental cost. Given the critical importance of roads and the current need to build them, the main countries involved in such infrastructure development stipulate regulations to mitigate environmental impacts throughout a road’s life cycle [4,5].

The ideal solution—building high-quality structures while reducing environmental impact—requires techniques that are less damaging to the environment, including designing less-polluting processes for mixing the asphalt, and using waste from other industries in the development of road infrastructure [6]. All of these ideas are inherent in the so-called “circular economy,” a strategy to reduce consumption of virgin materials, optimize industrial processes, and ultimately reduce waste. The circular economy enables industries to close economic and ecological flows of resources.

Based on the above, and following the principles of the circular economy, a trend toward less-polluting techniques with a similar quality of results is essential for preserving road infrastructure. One of the best solutions is the recycling of asphalt pavement with bitumen emulsion.

Road pavement made with an asphalt mix has good strength, adhesion, and comfort for drivers, but these characteristics can decrease significantly at the end of its useful life. However, the aggregates’ standardized and binding characteristics give the aged material quality for use in new asphalt mixes. Pavement aging is characterized by the formation of cracks of irregular shape and the loss of macroscopic adhesion for vehicles. Merely disposing of the aged pavement into landfill without any reuse wastes significant amounts of material, which is a strong negative environmental impact [7,8]. Ideally, mixtures would be developed that use this aged material to achieve the qualities required in the construction of the new infrastructure, reducing the use of new virgin materials in the construction of infrastructure, and of fossil fuels to produce and transport the new mixture [9,10]—in short, a lower emission of greenhouse gases and lower environmental impact. Some authors call this result sustainable pavement, thanks to the increase in the useful life of the infrastructure, and savings in terms of economic and environmental costs [11,12,13].

Lack of sustainability is a problem in the construction sector, albeit one which may slightly be mitigated through the reclamation of aged pavement to create a new asphalt mix. Disposing of the aged layer in landfill and producing a new asphalt mix should be rejected as an idea, due to its high impact on the environment and the contemporary imperative to optimize resources.

Various techniques have been used in this process. Every technique has its advantages and disadvantages, but each use all or part of the milled and aged road material.

These techniques are classified into two main types: hot and cold, depending on whether or not the asphalt mix is heated. In hot in-plant recycling, the road’s aged asphalt mix is milled, transported to the manufacturing plant, mixed with virgin material, and manufactured at temperatures of 180 °C. It is then transported to the site of the infrastructure construction project, spread, and compacted. Unlike hot recycling, cold recycling is performed at room temperature, reducing the consumption of fossil fuels and the emission of CO_2_ required in the heating of the mixture. Cold central plant pavement recycling-based processes follow implementation stages similar to those of hot recycling. One difference is the much lower temperature, cold in-place recycling does not require transportation of the milled mixture to a manufacturing plant, and the new mix is manufactured with 100% of the aged pavement reused in the new infrastructure [14]. 

Cold in-place recycling, on which this study is based, has a number of obvious advantages over other techniques. Environmental advantages include the reduced transport by heavy vehicles, lower CO_2_ emissions, and a lower consumption of fossil fuels. Operational advantages include [15,16] low traffic influence, the maintenance of road geometry, a high pace of construction, and safety. These advantages stem mainly from the efficient practices involved in the execution of all pavement manufacturing operations, from milling and mixing with emulsion, water, and other additives to spreading and compaction.

Based on the above, cold in-place recycling with bitumen emulsion decreases the environmental impact, mainly due to a reduced extraction of virgin materials, and less use of machinery in the transport and manufacture of the asphalt mix. These improvements significantly reduce the emission of greenhouse gases such as CO_2_ and NO_X_ [17].

Recycling pavement also has some disadvantages that must be taken into account during design. Firstly, not all pavements can be recycled; some pavements have suffered plastic deformation and are not recommended for regeneration, as the problems are likely to re-impact the new pavement created. Furthermore, since the new recycled pavement with bitumen emulsion is manufactured, curing time is required to achieve optimal mechanical properties. Therefore, even if it is permissible to open the road to traffic after the reclamation of the pavement, sufficient time is required before the final mechanical characteristics are obtained [18]. It is therefore essential to choose a bitumen emulsion compatible with both the material to be treated, and the expected breaking and curing times.

Secondly, the difficulty of reproducing the final characteristics of the projected pavement in the laboratory [19,20], variability of materials, and the dependence on proper execution make it difficult to determine the final behavior of the cold in-place recycling mix in advance, thus limiting its use to low traffic-volume roads [21].

In several cases, however, proper laboratory study and accurate execution of the technique has led to the creation of pavement with characteristics notably better than expected, producing a sustainable mix with good mechanical characteristics, even for high traffic [22,23,24]. Additives such as cement [25], and even industrial by-products such as fly ash from coal-fired thermal plants [26], have been used with good results.

Based on the preceding research, obtaining optimal mechanical characteristics in cold in-place recycling with bitumen emulsion requires the incorporation of an additive with cementitious characteristics to achieve three goals: proper pavement strength during the curing time; granulometric adjustment of the reclaimed asphalt pavement; and material that maximizes final resistance of the mix. This project therefore uses ladle furnace slag as a grading corrector and additive to improve mechanical qualities in the short and long term.

The ladle furnace slag comes from the steel industry. It is a by-product of the process of obtaining quality steel from scrap steel—more specifically, from the refining stage. In the first stage of meltdown performed in an electric arc furnace, oxidation eliminates manganese and silicon and achieves dephosphorization, creating a foamed slag in which all the dross accumulates. These slags are called electric arc furnace slags. The subsequent refining stage is for the removal of metal oxides, desulfurization, and decarbonization of the steel [27]. The main purpose of the refining stage is to obtain steel with a low oxygen and sulfur content, so the refining furnace or ladle furnace is fed with melted liquid from the previous stage and then covered with a reduction slag that is formed of lime, fluorspar, coke or graphite in appropriate amounts. Deoxidation is achieved by simple contact of the molten liquid with the slag. Full deoxidation occurs, however, through the addition of silicon and manganese ferroalloys, leading to liquid particles retained in the slag forming in the metal bath. Desulfurization minimizes the amount of sulfur in steel thanks to the presence of calcium oxide and carbon. This process produced the ladle furnace slag used in this project in a portion of 20–30 kg per final ton of steel. Although the proportion is lower than that obtained in the production of electric arc furnace slag, the landfill of ladle furnace slag produces serious environmental problems due to its chemical composition and small particle size.

Unlike electric arc furnace slags, with resistant characteristics, angularity, and hardness making them ideal substitutes for virgin aggregate in road infrastructure diversity (mainly in asphalt mix), ladle furnace slag has not been reused abundantly in the creation of new materials [28,29,30,31]. Ladle furnace slags have a fine grading which is suitable for various purposes [32,33]—among them, the addition to reclaimed asphalt pavements for the execution of cold in-place recycling with bitumen emulsion, the focus of this project. The presence of some metal oxides can cause volumetric expansion of the material when hydrated, however, and laboratory monitoring was required during the study [34,35].

Among the few applications of ladle furnace slag is its addition to cement [36,37], as a substitute for sand in cement mortars [38,39,40,41], as a replacement in concrete [42], and as a stabilizer of clayey materials due either to its high lime content [43] or its treatment of water [44,45]. Its appropriateness as a material with cementitious characteristics has been confirmed by the success stories involving its addition to cements, mortars, and concretes.

On the other hand, the use of ladle furnace slag in pavements has been limited, and only practiced at the research level. In the incorporation of the slags into low quality soils for its stabilization [46], acceptable results were obtained, with no problems of expansiveness in the treated material, and an improved quality of the soil. In turn, ladle furnace slags were used for the manufacture of concrete pavements [47], reflecting adequate strengths after testing, and even used as road bases, or subbases [48]. In the field of hot mix asphalt, research has been carried out in which the filler was replaced by ladle furnace slag [49], obtaining good properties of rigidity, tensile splitting strength and resistance to repeated loads. At the same time, the substitution of a high proportion of calcareous aggregates by slags in warm mix asphalt [50] showed a considerable improvement in mechanical properties compared with a traditional bituminous mix. Finally, the development of porous asphalt with ladle furnace slag and bitumen [51] showed excellent adhesion of the bitumen with the slag, resulting in good mechanical properties. However, there is no research referring to the use of ladle furnace slag in bituminous mixtures with bitumen emulsion and reclaimed asphalt pavements (RAP), the process developed in this research.

Based on the above, this study analyzed cold in-place recycling with bitumen emulsion and the incorporation of ladle furnace slag as an additive to improve grading and to provide special characteristics of resistance. The study therefore explored the use of industrial by-products as a substitute for virgin material, and also to lengthen the life cycle of a pavement. In addition to reducing the use of virgin materials in the formation of the asphalt mix, use of cold in-place techniques significantly decrease greenhouse gas emissions and fossil fuel consumption. This project sought to optimize the use of resources and manufacturing techniques, while reducing industrial waste. The study was therefore performed within the framework of a circular economy.

Regulations for studying the suitability of cold in-place recycling not only vary by country, but are somewhat inaccurate and depend on the empirical conditions of sampling, compaction, and testing [52]. Given the proliferation of such techniques in Spain for over 30 years, and the success achieved in their implementation, this study follows the Spanish regulations outlined in Circular 8/2001 [53], which provides a series of warnings concerning the treatment of materials and a series of standardized tests to be performed to achieve results that conform to the various minimum values established. 

This regulation was applied for the in-place implementation of a bituminous mixture with bitumen emulsion, ladle furnace slag and milled pavement as the most superficial layer. Subsequently, a bituminous mixture reduced in thickness was applied on this pavement, which would improve the friction of a tyre with the road and provide a comfortable and safe wearing layer.

## 2. Materials and Methods 

This section details the starting materials and the methodology used to study the suitability of in-place recycling of asphalt pavement with bitumen emulsion, using ladle furnace slag as an additive.

### 2.1. Materials

The following subsections detail the materials used in this study, highlighting their nature and origin, as well as specific noteworthy characteristics.

#### 2.1.1. Reclaimed Asphalt Pavements

The reclaimed asphalt pavement came from the surface layer of the road joining the towns of Linares and Jabalquinto, located in Spanish territory. The road on which the pavement was located had a medium volume of heavy-vehicle traffic, and the surface layer was cracked with an irregular shape. This cracking reflected structural depletion of the layer due to aging of the bitumen. The layer did not, however, show significant deformation due to poor design of the initial mix or poor execution of the subgrade. The pavement composed of hot mix asphalt was milled with machinery similar to that which would be used on-site, to obtain an appropriate sample for study. The tests performed to confirm the pavement’s suitability for recycling are detailed in the methodology.

#### 2.1.2. Bitumen Emulsion

Bitumen emulsion plays a key role in achieving the mechanical and durability characteristics of the final asphalt mix. As an element to bond the various particles of the reclaimed asphalt pavements and the ladle furnace slag, bitumen emulsion can provide the asphalt mix with both tensile strength and durability.

Choosing the right bitumen emulsion was essential for a variety of reasons, including achieving adequate adhesivity between the aggregates and the bitumen emulsion, and breaking and coating times suitable for execution on-site. To achieve these properties, a slow-breaking bitumen emulsion must be used, since longer breaking time favors coating of the reclaimed asphalt pavements particles. To achieve chemical compatibility between aggregates, reclaimed asphalt pavements, and ladle furnace slag, a cationic emulsion with characteristics appropriate to the nature of ladle furnace slag was chosen; this emulsion did not cause problems in the coating of the reclaimed asphalt pavements. European regulations label this emulsion C60B5 REC. Table 1 lists its technical characteristics.

#### 2.1.3. Ladle Furnace Slag

The ladle furnace slag used in this study came from the steel manufacturing industry. As mentioned above, ladle furnace slag is found in steel refining or ladle furnaces. The sample was taken representatively, such that it contained all particle sizes typical of the unaltered by-product. The following sections detail the tests performed to characterize and study the by-product.

### 2.2. Methodology

A clear, objective methodology was used to confirm the suitability of the mechanical and resistance-related characteristics produced by cold in-place recycling with bitumen emulsion and ladle furnace slag.

Firstly, both of the starting materials, reclaimed asphalt pavement and ladle furnace slag, were analyzed to determine their physical, chemical, and mechanical characteristics. Consideration was given to differences in the materials, as well as to the role each played in the final asphalt mix. Individualized study best determined the critical characteristics that could pose problems in executing the job.

After analyzing the properties of the various materials and their suitability for use in asphalt pavement recycling, the different specimen families were manufactured. Firstly, however, the percentages of each material to be added—reclaimed asphalt pavement, bitumen emulsion, ladle furnace slag, and water—was determined.

It should be noted that the mixing sequence was as follows: first, the pavement was milled and added to the ladle furnace slag; secondly, the appropriate percentage of precoating water was incorporated to facilitate the mixing process; and finally, the bitumen emulsion was added by re-mixing to achieve a homogeneous mix of materials.

According to Spanish Circular 8/2001 [53], these percentages were determined through a series of steps outlined in the NLT-389/00 [66] standard. This regulation primarily describes the grading envelope for in-place recycling with bitumen emulsion. The grading curve obtained by combining different percentages of the recycled asphalt pavement and ladle furnace slag must be contained in the grading envelope. After defining the percentages of each material, the percentages of fluids (bitumen emulsion and pre-coating water) required for the final mix were identified. NLT-389/00 [66] establishes the margins for both materials, and the addition of fluids varied based on these margins. These margins are determined by the UNE 103,501 [67] Modified Proctor Compaction Test and the NLT-196/84 [68] test for bitumen coating. Only in this way could a final mix with the greatest resistance be created, due to the higher density determined by the Modified Proctor Compaction Test. Good mechanical characteristics depend on the coating and adhesion of the aggregates with the emulsion, which was quantified by the Coating Test.

After determining the percentage of each material to be added to obtain the different families, the same tests were performed: simple compression strength NLT-161/98 [69] and immersion-compression NLT-162/00 [70].

With the data obtained from dry and post-immersion resistance, as well as preserved resistance, the optimal formula for the job was calculated mathematically, providing a glimpse of the mechanical characteristics of this job mix formula to be verified later.

The following defines the steps detailed in each section.

#### 2.2.1. Analysis of Starting Materials

Industrial by-products have an environmental advantage over virgin materials, as using the former reduces the environmental impact by decreasing the extraction rate of other materials. These by-products must be studied in detail, however, as most have special characteristics that could cause revalorization to fail. 

Among the by-products used in this study, reclaimed asphalt pavement was studied to determine its physical, chemical, and mechanical characteristics. Firstly, particle size was analyzed using the test UNE-EN 933-1 [71], to assess suitability for the grading envelope stipulated by this standard, as well as the percentage of ladle furnace slag to be added. After analyzing particle size, the binder and coarse and fine aggregates were separated according to UNE-EN 12697-1 [72]. The aged binder was then studied through the UNE-EN 1426 [62] penetration and UNE-EN 1427 [63] softening point tests, to evaluate the aging point of the binder. Next, since the coarse aggregate of the reclaimed asphalt pavement is responsible for providing mineral skeleton to the mix, the following tests are compulsory: determination of resistance to fragmentation, UNE-EN 1097-2 [73]; determination of percentage of crushed and broken surfaces in coarse aggregate particles, UNE-EN 933-5 [74]; and flakiness index, UNE-EN 933-3 [75]. The fine aggregate was tested using the sand equivalent test UNE-EN 933-8 [76] and the plasticity index (UNE 103,103 [77] and UNE 103,104 [78]) to identify the presence of clay elements that could impair the mix.

Next, the ladle furnace slag was studied chemically and physically. An X-ray fluorescence test provided the elemental composition of the sample, enabling evaluation of its chemical aptitude for the final mix and expected cement characteristics, as well as chemical elements that could pose problems in the mixture once the road is finished.

The physical properties were then evaluated through particle size analysis, UNE-EN 933-1 [71], a determination of particle density, UNE-EN 1097-7 [79], and the bulk density of filler in kerosene, UNE-EN 1097-3 [80]. These typical civil engineering tests are essential in the study of waste, because variation from the density of the usual virgin material necessitates appropriate volumetric corrections. As with the fine aggregate, the material’s plasticity was analyzed (UNE 103,103 [77] and 103,104 [78]) to prevent it from impairing the final mix due to expansivity problems.

#### 2.2.2. Manufacture of the Different Sample Families and Tests

After analyzing the starting materials and evaluating their suitability for cold in-place recycling with bitumen emulsion, the corresponding grading curve was adjusted by adding the ladle furnace slag to the reclaimed asphalt pavement. The grading curve obtained by combining the two materials must conform to the grading envelope defined by Circular 8/2001 [53], more specifically RE2. The type of mix (RE2) was chosen for its reduced layer thickness, and thus its more suitable mechanical characteristics.

Once the combination percentage of reclaimed asphalt pavement and ladle furnace slag was determined, the percentages for the addition of fluids (bitumen emulsion and precoating water) were studied. Following the NLT-389/00 [66] standard, we first calculated the Theoretical Content of Fluids (TCF), that is, the percentage of optimal humidity obtained from the Modified Proctor Compaction test UNE 103,501 [67] for combining reclaimed asphalt pavement and ladle furnace slag. The value is the reference for the percentage of precoating water, plus the percentage of bitumen emulsion that must be added to the aggregates to achieve maximum density and approximate highest resistance.

The definitive percentage, termed Optimal Fluid Content (OFC), was determined by performing several coating tests with different percentages of precoating water, while keeping the emulsion percentage constant. This procedure should produce a total fluid content (water plus emulsion) ranging from TCF–2% to TCF. The coating test was performed in accordance with NLT-196/84 [68], adding no calcium carbonate and only the combined reclaimed asphalt pavement and ladle furnace slag, as well as the emulsion and precoating water stipulated. A coating test then evaluated the suitability of the emulsion in combination with the aggregates, enabling selection of the percentage corresponding to the Optimal Fluid Content (OFC). This value had to be between TCF–1% and TCF%.

Following the determination of the Optimal Fluid Content (OFC), the percentages of precoating water and bitumen emulsion for the different families were determined, based on the knowledge that the emulsion percentage should be within a range of 2.5–4%. Three families were created with different percentages of bitumen emulsion-to-aggregate (recovered asphalt pavement plus ladle kiln slag) of 3%, 3.5% and 4%. Each percentage of emulsion corresponded to a percentage of precoating water equal to the difference between the Optimum Fluid Content (OFC) and the percentages of emulsion. 

After determining the composition of the different families, 12 samples were manufactured for each family, designed according to the norm, NLT-161/98 [69]. The compaction process according to the detailed standard consisted of: first, pouring the bituminous mixture into standardized molds; next, an initial load of 1 MPa was then applied; finally, a load of 21 MPa was applied over a time of 2 to 3 min at constant speed. After manufacturing, these samples had to be cured in a forced air oven at 50 ± 2 °C until they reached constant mass, for no less than 3 days and not more than 7 days.

Upon completion of this process, maximum density, UNE-EN 12697-5 [81], and bulk density of the specimens, UNE-EN 12697-6 [82], as well as the void content, UNE-EN 12697-8 [83], of the different families was determined. The goal was to divide each family into two groups to study the effect of water on cohesion of the compacted asphalt mix (immersion-compression test NLT-162/00 [70]). A group from each family was subjected to the action of water to study cohesion. The samples were submerged in a water bath regulated at 49 ± 1 °C for 4 days and then tested following NLT-161/98 [69] to evaluate the difference in resistance between the sample subjected to the action of the water and the sample kept dry.

#### 2.2.3. Determination of the Optimal Job Mix Formula 

Once the results for densities, dry resistance, post-immersion resistance, and preserved resistance were obtained for the different families, they were evaluated mathematically and graphically to obtain maximum dry and post-immersion resistance results. These values had to be higher than the minimums stipulated in Circular 8/2001 [53]—a dry resistance value of 3 MPa and post-immersion resistance of 2.5 MPa. Once the maximum and expected resistance values were calculated by mathematical correlation, the specimen family was created with the optimal job mix formula in order to corroborate the approximate values. Ultimately, once resistance was confirmed, this step yielded the ideal combination of reclaimed asphalt pavement, ladle furnace slag, bitumen emulsion, and water to manufacture cold in-place recycled pavement.

It should be noted that the optimum combination of materials was obtained through the dry resistance test because it is the test that is most limited by the regulations, and which also best characterizes the bituminous mixture, as it provides the resistance of this structural course.

## 3. Results and Discussions

The following subsections present the results of the methodology described above.

### 3.1. Results of the Analysis of the Starting Materials 

**Figure 1:**
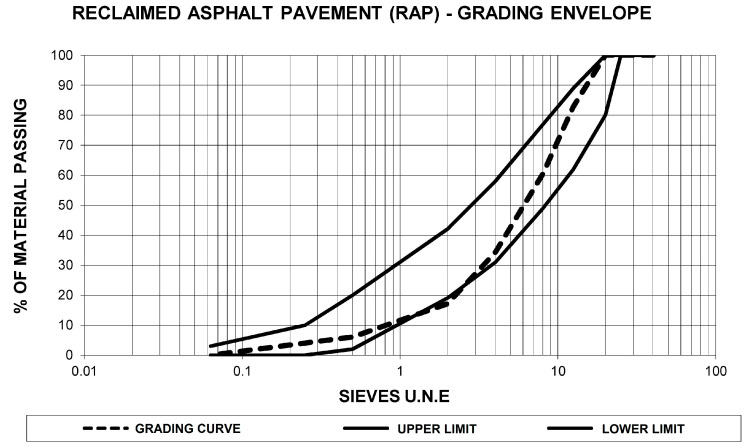
Graph of the grading curve of reclaimed asphalt pavement referenced to the RE2 grading envelope of Circular 8/2001 [53].

Particle size of the reclaimed asphalt pavement was analyzed to study its grading curve. This analysis enabled the adjustment of the percentage of ladle furnace slag added, to comply with the grading envelope established in the regulations. Figure 1 displays the results of the particle size analysis and the correspondence of the particle size distribution obtained for the reclaimed asphalt pavement to the RE2 grading envelope stipulated by Circular 8/2001 [53].

As Figure 1 shows, the grading curve of the reclaimed asphalt pavement reflects a majority composition of coarse aggregate, with a maximum size of less than 20 mm. Furthermore, the low proportion of fine aggregate led to the addition of ladle furnace slag adjusting the grading curve to the established grading envelope, as well as to reveal its cementitious characteristics. 

Once the binder was extracted from the reclaimed asphalt pavement, separation of the aggregate enabled determination of the existing amount of the binder in the aggregate as 4.3%. This percentage is common in semi-dense asphalt mix and continuous grading used for medium- or low-traffic roads. The extracted bitumen binder was then analyzed (see Table 2 for results).

The results for penetration and softening point confirmed expectations. The pavement had aged by exhaustion, causing the binder to mostly lose its elasticity and become excessively hard. It should be noted that the hardness values were also influenced by the type of bitumen used in the area from which the pavement was reclaimed, where very hard bitumen (type B 40/50) is used due to the warm climate.

Moreover, the aggregate extracted from the pavement, unmilled to avoid alteration, was analyzed to confirm its suitability. The results of the tests are detailed in Table 3.

The results shown in Table 3 reflect a coarse aggregate of acceptable mechanical resistance and particle shape, more than adequate for use as aggregate in an asphalt mix for roads with intermediate traffic volume. The low plasticity index and sand equivalent value greater than 75 rule out the possible presence of clay particles that could cause problems due to expansiveness.

In line with the previous assumptions, reclaimed asphalt pavement can be classified as suitable for use in cold in-place recycling, but not without first correcting the grading through the addition of ladle furnace slag, and studying its compatibility with bitumen emulsion.

The ladle furnace slag was then analyzed elementally to detect its composition and to identify elements that could cause problems. Table 4 presents the results of the X-ray fluorescence.

The results show that the ladle furnace slag’s composition derives directly from its nature and the production process, highlighting the percentages of calcium oxide so necessary for achieving primary resistance of the shaped mixture, and thus its suitability for supporting traffic. Percentages of silicon oxides are also necessary to achieve good cementitious characteristics over time.

**Figure 2:**
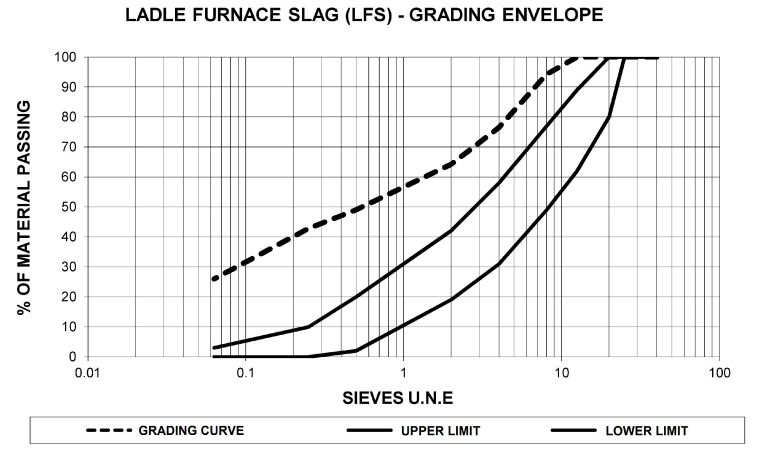
Graph of the grading curve of ladle furnace slag referenced to the RE2 grading envelope of Circular 8/2001 [53].

The grading of the ladle furnace slag obtained in the physical tests is presented in Figure 2.

The results show a significant percentage of fine aggregates and a lower proportion of coarse aggregates, with a maximum aggregate size of 12.5 mm.

The remaining physical tests of density and plasticity for the fine portion of the ladle furnace slag are detailed in Table 5.

Density values did not vary from those of typical virgin material filler; they showed a bulk density value of less than 0.8 t/m^3^, indicating non-pulverulent behavior that would not impair operability. Plasticity is clearly zero, as this material had a significant percentage of calcium oxide and silicon oxide.

Therefore, after the study of both materials, reclaimed asphalt pavement and ladle furnace slag, it can be concluded that both are suitable for use in new bituminous mixtures. The reclaimed asphalt pavement had aged bitumen, albeit with an acceptable quality of aggregate for its reuse, while the ladle furnace slag had a chemical composition suitable for the development of the expected cementitious characteristics, and a fine grain size ideal for combination with the reclaimed asphalt pavement.

### 3.2. Test Results of the Different Sample Families

**Figure 3:**
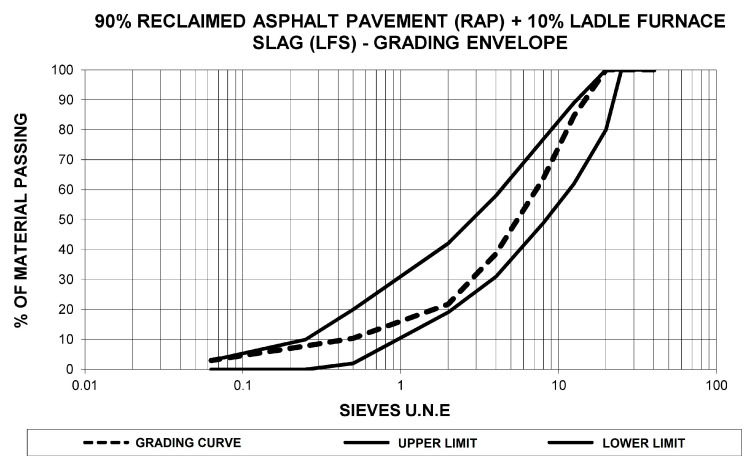
Graph of the grading curve for the combination of 90% reclaimed asphalt pavement and 10% ladle furnace slag, referenced to the RE2 grading envelope of Circular 8/2001 [53].

Based on the particle size analysis of the reclaimed asphalt pavement and ladle furnace slag, the percentage of each element to be added was calculated to confirm achievement of the grading envelope stipulated in Circular 8/2001 [53], and to incorporate an adequate percentage of ladle furnace slag to produce the cementitious characteristics that make the pavement resistant. The proportion of elements in the combination was 90% reclaimed asphalt pavement and 10% ladle furnace slag, a percentage chosen based on detailed adjustments. Figure 3 displays the corresponding grading curve for the mixture of the two materials. In the following sections, aggregate will be used to indicate the combination of these materials in the percentages specified, with the bitumen emulsion and water percentages referring to the mass of the two together.

Once the percentage of the combined materials was determined, the Modified Proctor Compaction Test UNE 103,501 [67] was performed to determine the optimal humidity to obtain maximum compaction density. Optimal humidity, also termed the Theoretical Content of Fluids (TCF), corresponds to precoating water plus emulsion.

**Figure 4:**
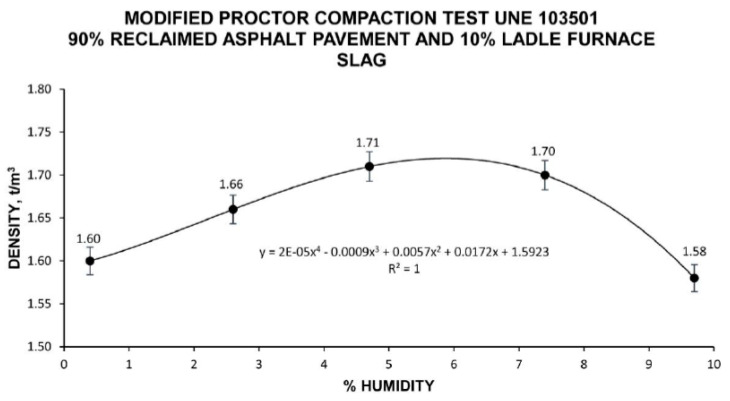
Graph of the Modified Proctor Compaction Test UNE 103,501 [67] for the combination of 90% reclaimed asphalt pavement and 10% ladle furnace slag.

The Modified Proctor Compaction Test was performed for water percentages of 0%, 2.5%, 5%, 7.5%, and 10% in the mixture, establishing a clear maximum density value (1.72 t/m^3^) with a humidity of 5.9%. The results of the Proctor Test can be seen in Figure 4.

**Figure 5:**
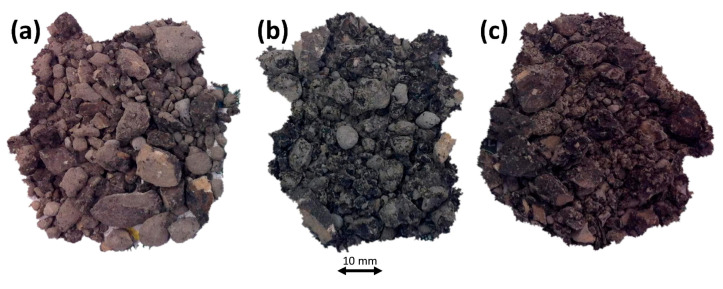
Coating Test NLT-196/84 [68] for reclaimed asphalt pavement, ladle furnace slag, and 3% emulsion with different percentages of precoating water. (**a**) 0.9% water-to-aggregate. (**b**) 1.9% water-to-aggregate. (**c**) 2.9% water-to-aggregate.

After determining the Theoretical Content of Fluids (TCF) (5.9% to aggregate), Optimal Fluid Content (OFC) was calculated, starting with an essential test, the coating test NLT-196/84 [68]. An emulsion percentage of 3% to aggregate (reclaimed asphalt pavement plus ladle furnace slag) was established, as well as increasing percentages of precoating water from TCF–2% to TCF—that is, 0.9%, 1.9%, and 2.9% water-to-aggregate. Figure 5 presents images of adhesion of the emulsion with different percentages of precoating water.

Examination of the Coating Tests identified the best adhesion and coating of the emulsion, which occurred in the mix with 2.9% precoating water and 3% emulsion-to-aggregate, and was classified as good. This material’s good dry behavior was easily observable, as it coincided in this case with the Theoretical Content of Fluids (TCF). It should be noted that the wrapping times were less than 60 s, and the breaking times of the emulsion were less than 300 s, reliable proof of compatibility of the emulsion with the aggregate.

Once Optimal Fluid Content (OFC) was determined, the different families of samples were manufactured with increasing percentages of emulsion. Circular 8/2001 [53] establishes a percentage of emulsion for cold in-place recycling of 2.5–4% to aggregate. To cover the entire possible range of combinations, therefore, three families were made, with increasing percentages of emulsion in increments of 0.5%, from 3% to 4% emulsion-to-aggregate. The 2.5% emulsion family was eliminated based on the assumption that this percentage of emulsion would be insufficient due to the large quantity of fine particles present. The precoating water for each family was calculated as the difference between Optimal Fluid Content (OFC) and the corresponding emulsion percentage. The percentage additions of precoating water and emulsion for each family are displayed in Table 6.

In total, 12 specimens were manufactured for each group following the standard NLT-161/98 [69]. After curing to a constant mass at a temperature of 50 ± 2 °C in a forced air stove, the specimens from each family were subdivided into two groups, which were subjected to different conditions. One group was immersed in water and the other kept dry, to assess the effect of water on the cohesion of the bituminous mixture. Table 7 shows the average values of particle density, bulk density, void content, dry compressive strength, post-immersion compressive strength, and preserved resistance.

Based on the minimum values set forth in the Spanish regulations (3 MPa for dry resistance and 2.5 MPa for post-immersion compressive resistance), the values of 3% emulsion and 2.9% precoating water were acceptable in principle, as were 3.5% emulsion and 2.4% of precoating water-to-aggregate. The values obtained showed a clear decrease in the index of voids in the mixture with a higher percentage of emulsion, due to the compactability conditions provided by the emulsion. Based on dry compressive strength, the results identified an optimum point of 3–3.5% of emulsion-to-aggregate, as well as a decrease in post-immersion resistance with higher percentages of emulsion. The mathematically calculated optimal job mix formula provided maximum strength based on the results analyzed, showing the optimum combination of reclaimed asphalt pavement, ladle furnace slag, bitumen emulsion and water.

### 3.3. Optimal Job Mix Formula

**Figure 6:**
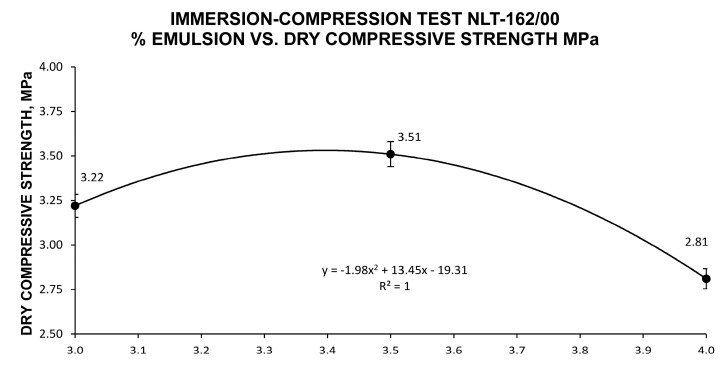
Test of dry compressive strength, NLT-162/00 [70], for the different emulsion percentages of each family of specimens.

Once the different specimen families were evaluated, the percentage of emulsion to obtain maximum resistance was studied mathematically. This percentage depended on the dry resistance and the points obtained from the different families. The maximum of this function, illustrated in Figure 6, coincided with 3.3% emulsion-to-aggregate and 2.6% precoating water.

Using the maximum obtained mathematically, and the percentages of the different materials to be added, 12 specimens were manufactured to confirm the optimal properties obtained with this job mix formula.

The process of manufacturing, curing, and studying the effect of water was similar to that performed on the other families. Table 8 presents the results of the trial for this optimal job mix formula.

The combination of 90% reclaimed asphalt pavement to 10% ladle furnace slag, plus 2.6% precoating water and 3.3% emulsion, produced results superior to those of the other families. The values for dry compressive strength, post-immersion compressive strength, and preserved resistance were higher than those required by the relevant regulations. The minimum values established by Circular 8/2001 [53] are 3 MPa for dry compressive strength and 2.5 MPa for post-immersion compressive strength, with a Preserved Resistance Index maintained as greater than 75%.

The following should be highlighted: a high percentage of RAP was utilized. This percentage of 90% RAP would have been unacceptable in other techniques that do not use bitumen emulsion and just use bitumen (e.g., hot mixes asphalt). This percentage of RAP was adequate, as reflected in the compressive strength tests. These results were obtained thanks to its cementitious properties and by the addition of ladle furnace slag. Finally, it should be pointed out that the same milling equipment was used for the laboratory tests as would later be used in the execution of the bituminous mix, since otherwise the particle size distribution could vary.

## 4. Conclusions 

The following summarizes the partial conclusions that can be drawn from the results of the tests described in the methodology:

- Elementary composition of the ladle furnace slag studied showed a majority percentage of calcium oxide and a lower percentage of silicon oxide. Both compounds are essential for developing the desired cementitious characteristics of the ladle furnace slag and providing the strength of the asphalt mix manufactured with them.
- The ladle furnace slag had a maximum aggregate size of less than 12.5 mm, with primarily fine grading. Its particle and bulk density were comparable to those of a conventional aggregate and did not show plasticity.
- The slow-breaking cationic bitumen emulsion C60B5 REC showed good compatibility with the combination of ladle furnace slag and reclaimed asphalt pavement, as shown by the coating test. Both the adhesion of the emulsion to the aggregate and its cohesion and breaking times are suitable for use with ladle furnace slag and reclaimed asphalt pavement, with maximum precoating amounts of water-to-aggregate of 2.9% and emulsion-to-aggregate of 3%.
- The tests of both simple dry and post-immersion compressive strength showed good results (superior to Spanish regulations) for 3–3.5% emulsion-to-aggregate, corresponding to 2.9–2.4% precoating water-to-aggregate, respectively.
- The optimal combination of the different materials—considering an aggregate mixture of 90% reclaimed asphalt pavement and 10% ladle furnace slag, emulsion percentages of 3.3% to aggregate, and 2.6% precoating water—showed values of simple dry compressive strength and post-immersion compressive strength higher than those established by Spanish regulations and those of the other families tested.

Based on these detailed partial conclusions, ladle furnace slag has good characteristics for manufacturing pavement through cold in-place recycling with bitumen emulsion. The addition of ladle furnace slag achieved, on the one hand, the appropriate adjustment of the particle size of the reclaimed asphalt pavement, and on the other hand, provided resistance characteristics observable in the results obtained. It was therefore an ideal solution that created a sustainable asphalt mix: by considerably reducing CO_2_ emissions; that used by-products in its composition therefore avoiding their disposal in landfills; and that was in-keeping with new environmental and circular economy trends.

## Figures and Tables

**Table 1:** Technical details of the bitumen emulsion C60B5 REC.

Characteristics	Unit	Standard	Min.	Max.
**Original Emulsion**				
Particle polarity	-	UNE EN 1430 [54]	Positive	
Breaking value	g	UNE EN 13075-1 [55]	170	
Binder content (per water content)	%	UNE EN 12846-1 [56]	58	62
Efflux time (2 mm, 40 °C)	s	UNE EN 12,846 [57]	15	70
Residue on sieving (0.5 mm)	%	UNE EN 1429 [58]	-	0.10
Setting tendency (7 days storage)	%	UNE EN 12,847 [59]	-	10
Water effect of binder adhesion	%	UNE EN 13,614 [60]	90	-
**Binder after Distillation (UNE EN 1431** [61])				
Penetration (25 °C; 100 g; 5 s)	0.1mm	UNE EN 1426 [62]	-	270
Softening point	°C	UNE EN 1427 [63]	35	-
**Evaporation Residue (UNE EN 13074-1** [64])				
Penetration (25 °C; 100 g; 5 s)	0.1mm	UNE EN 1426 [62]	-	330
Softening point	°C	UNE EN 1427 [63]	35	-
**Stabilizing Residue (UNE EN 13074-2** [65])				
Penetration (25 °C; 100 g; 5 s)	0.1mm	UNE EN 1426 [62]	-	270
Softening point	°C	UNE EN 1427 [63]	35	-

**Table 2:** Tests of binder extracted from reclaimed asphalt pavement.

Test	Standard	Value/Unit
Penetration (25 °C; 100 g; 5 s)	UNE-EN 1426 [62]	7 ± 0 (1/10) mm
Softening point	UNE-EN 1427 [63]	88 ± 2 °C

**Table 3:** Tests of the coarse aggregate and fine aggregate of the reclaimed asphalt pavement.

**Coarse Aggregate**		
**Test**	**Standard**	**Value/Unit**
Determination of percentage of crushed and broken surfaces	UNE-EN 933-5 [74]	93 ± 2%
Flakiness index	UNE-EN 933-3 [75]	88 ± 2 °C
Los Angeles Test method	UNE-EN 1097-2 [73]	19 ± 1%
**Fine Aggregate**		
**Test**	**Standard**	**Value/Unit**
Plasticity index	UNE 103103/UNE 103,104 [77,78]	2.9 ± 0.1%
Sand equivalent	UNE-EN 933-8 [76]	79 ± 2%

**Table 4:** Results of the X-ray fluorescence of ladle furnace slag.

Compound	wt,%	Est. Error
CaO	40.19	0.25
MgO	19.38	0.20
SiO_2_	12.49	0.17
Al_2_O_3_	7.29	0.13
Fe_2_O_3_	2.38	0.08
MnO	0.936	0.047
S	0.548	0.027
TiO_2_	0.486	0.024
BaO	0.240	0.012
Na_2_O	0.118	0.042
Cr_2_O_3_	0.1100	0.0055
Cl	0.0833	0.0042
SrO	0.0733	0.0037
ZnO	0.0681	0.0034
K_2_O	0.0506	0.0025
ZrO_2_	0.0425	0.0021
V_2_O_5_	0.0179	0.0017
P	0.0138	0.0012
CuO	0.0117	0.0010
NiO	0.0082	0.0011
PbO	0.0048	0.0010
Nb_2_O_5_	0.0046	0.0006
MoO_3_	0.0028	0.0009
Co_3_O_4_	0.0021	0.0009
SeO_2_	0.0012	0.0005

**Table 5:** Density and plasticity tests for the fine portion of ladle furnace slag.

Test	Standard	Value/Unit
Particle density	UNE-EN 1097-7 [79]	2.71 ± 0.07 t/m^3^
Bulk density	UNE-EN 1097-3 [80]	0.75 ± 0.01 t/m^3^
Plasticity index	UNE 103,103/UNE 103,104 [77,78]	No plasticity

**Table 6:** Group of specimens manufactured with different percentages of emulsion and precoating water-to-aggregate.

Group	1	2	3
Precoating water-to-aggregate, %	2.9	2.4	1.9
Emulsion-to-aggregate, %	3	3.5	4

**Table 7:** Test of maximum density, bulk density, void content, dry compressive strength, post-immersion compressive strength, and preserved resistance for the different groups of specimens.

Test	Standard	1	2	3
Maximum density, t/m^3^	UNE-EN 12697-5 [81]	2.34 ± 0.04	2.31 ± 0.03	2.31 ± 0.08
Apparent density, t/m^3^	UNE-EN 12697-6 [82]	2.14 ± 0.08	2.15 ± 0.07	2.14 ± 0.04
Void content, %	UNE-EN 12697-8 [83]	8.37 ± 0.31	6.81 ± 0.15	7.04 ± 0.25
Dry compressive strength, MPa	NLT-162/00 [70]	3.22 ± 0.12	3.51 ± 0.12	2.81 ± 0.09
Immersion compressive strength, MPa	NLT-162/00 [70]	2.90 ± 0.04	2.63 ± 0.03	1.99 ± 0.03
Preserved Resistance Index, %	NLT-162/00 [70]	90 ± 2	75 ± 2	71 ± 1

**Table 8:** Tests for family of specimens made with the optimal job mix formula: 3.3% emulsion and 2.6% precoating water-to-aggregate.

Optimal job mix formula		
Test	Standard	Value/Unit
Precoating water, % of aggregate	-	2.6
Emulsion, % of aggregate	-	3.3
Maximum density, t/m^3^	UNE-EN 12697-5 [81]	2.32 ± 0.07
Bulk density, t/m^3^	UNE-EN 12697-6 [82]	2.15 ± 0.04
Void content, %	UNE-EN 12697-8 [83]	7.30 ± 0.27
Dry compressive strength, MPa	NLT-162/00 [70]	3.65 ± 0.06
Immersion compressive strength, MPa	NLT-162/00 [70]	2.91 ± 0.08
Preserved Resistance Index, %	NLT-162/00 [70]	80 ± 2
